# Supplementary material for: Demographic differentials of lung cancer survival in Bangladeshi patients
Source: PLoS One. 2021 Dec 10;16(12):e0261238. doi: 10.1371/journal.pone.0261238 (PMC8664208; doi:10.1371/journal.pone.0261238)
Supplement: Attachment — Submitted filename: Response to Reviewers.docx [file pone.0261238.s001.docx]

2^ND^ October, 2021

Wen-Wei Sung, M.D., Ph.D.
Academic Editor
PLOS ONE

**Reference:** PONE-D-21-18895 [Article Title: Demographic differentials of lung cancer survival in Bangladeshi patients]

Dear Professor Wen-Wei Sung,

Thank you so much for providing both the opportunity to resubmit as well as the thoughtful comments provided by all the reviewers. We are thankful to the reviewers for the insightful comments and directives on our submitted manuscript. We believe these comments are invaluable and will significantly raise the quality of this manuscript. We put our best effort to accommodate the comments and revise the manuscript accordingly. To assist reading this document, we put our response in blue text and all the texts added in the revised manuscript are presented with bright purple text.

| **Comments** | **Author Responses** |
| --- | --- |
| **Reviewer 1** | |
| MAJOR: This is purely a descriptive study of lung cancer. While classic histology was used to characterize the lung cancer types, it would be worthwhile to know if any mutation analysis was available that could identify genetic drivers of lung cancer. This may well contribute to prognosis irrespective of radiotherapy or chemotherapy. Tobacco exposure seems to be a major factor in lung cancer occurrence. But in cases where it was absent, can the authors speculate on potential non-tobacco related causes? Such as environmental exposure to organic or inorganic dusts, air pollution, smog, indoor smoke exposure from burning fires for cooking. The data are provocative in the sense that it is from a resource limited country where such reporting is often limited so the data are useful to know. It is from a single centre although it is a central referral site for cancer evaluation which means people with more advanced disease are likely to be referred so there may be a selection bias toward poorer outcomes.  It would be useful to know how decision were made to provide radiotherapy or chemotherapy or both to individual cases. Small cell cancer is treated with chemotherapy and adenocarcinomas are relative resistant to radiotherapy and chemotherapy. Can this be included in the report? | Many thanks for the comment. We agree with the reviewer about the importance of the mutation analysis. Unfortunately, National Institute of Cancer Research and Hospital (NICRH), despite being the apex referral cancer centre in the country, is not yet equipped with the facility and capacity of mutation analysis, hence we could not provide any insight in this regard.  We have included the following text in the **discussion** section of the manuscript accordingly    Being single centre is a limitation of the study, we acknowledged this as limitation. Regarding selection bias the reviewer have raised a very legitimate and important concern. However, the main factor that would contribute to the selection bias is the socioeconomic status, rather than the stage of the disease.  “Patients recruited from the government-funded hospital are likely to exclude a large section of well-off patients of high SES strata, who preferred private hospitals over the public-funded hospital, making the patient population in the study, poorer than the actual Bangladeshi patient population. To keep the selection bias to its minimum and to ensure capturing the spectrum of disease severity, we recruited all the consenting patients with complete data, attended the medical oncology department during the study period.”  Regarding choice of treatment, we agree with reviewers’ comment. Following text has been added in the method section regarding choice of treatment.  “After complete assessment of the patient, Histology based standard chemotherapy and radiotherapy protocols guided by the National Comprehensive Cancer Network (NCCN) guidelines were followed by an institutional multidisciplinary tumor board (comprise medical Oncologist, Radiation oncologist, surgical oncologist, radiologist and pathologist) for both curative and palliative setting. |
| **Minor**  1) English usage and spelling will need to be amended throughout the manuscript. It is unacceptable in its present form. 2) Please clarify the exclusion of patients with targeted therapy immunotherapy. Where there no such patients or was the information on them incomplete so unavailable for study? 3) Squamous cell and small cell cancers are classic tobacco related cancers but adenocarcinomas were the second most common lung cancer type.  Was adenocarcinoma more common in woman? Was tobacco exposure more common in men? Any speculation on the frequency of adenocarcinomas? 4) There is little comment on the presence of metastatic disease at the time of diagnosis or a part of therapy. Can this be included? | Thank you so much for the comment.  1.We have now made our best effort to review the English usage and spelling.  2. We acknowledge, our patient population did not include patient with targeted therapy. Unfortunately, National Institute of Cancer Research and Hospital (NICRH), despite being the apex referral cancer centre in the country, is not yet equipped with the facility and capacity of several targeted therapy, immunotherapy in particular. We have added the following text in the revised manuscript to address reviewer’s comment.  “despite being the apex referral cancer centre in the country, NICRH is not yet equipped with the facility and capacity of targeted therapy, immunotherapy in particular. As a result, our patient population did not include patients with targeted therapy (i.e. immunotherapy).  3. Thanks for the insight, we have revealed the following information from the data and added relevant text in the **discussion** section of the revised manuscript.  Prevalence of Adenocarcinoma is higher in female (46.3%) than male (36.9%), and conversely the prevalence of Squamous cell carcinoma is higher in male (43.2%) than female (37.5). The finding supports the classical conjuncture of squamous cell lung cancer being related to tobacco exposure. Gender specific tobacco exposure also support the fact, as tobacco consumption was higher among males (85.4%) in comparison to female (55.6%).  4.Thanks for the comment, this is important, and we have included the following text in the **result** section.  “About 19.4% patients had metastatic disease during the presentation. A third of those patients had liver metastasis.” |
| Reviewer #2 |  |
| This is an important study which shows findings in a developing country. It has noted important determinant factor such as education which is important to highlight. -Education and socio-economic status are closely associated with outcomes even in developed country but in developing country the difference becomes rather stark. -This study did not find any major difference in mortality based on socioeconomic status was surprising but the reason for that was well explained in the discussion. | Many thanks for your comment. This is a huge encouragement for us. |
| Reviewer #3 |  |
| I greatly appreciate the importance of studying the demographic features of survival in patients with lung cancer, especially since it is a study that involved such a large number of participants. The article is well written with very few grammatical errors Abbreviations must be mentioned at the bottom of the tables | Thanks for the valuable comments.  1. We put our best effort to correct the grammatical errors  2. We have added the abbreviations at the bottom of the tables in the revised manuscript. |
| Reviewer #4 |  |
| 1) Lack of background explanation on parameter representing performance status.  1a. The authors use the ECOG score to stratify performance status. While they do give the full name for ECOG [Eastern Cooperative Oncology Group] in the Materials & Methods section, they do not give the full name in the abstract nor in the table 1 legend and they should add it to these areas.  1b. The authors only state that the ECOG score is from 0 to 4 and ultimately placed this cohort int 2 groups with a cut point score of 2 or more. The authors should consider stating what the scores correspond to [i.e., a sore of 0= fully active and score of 4= completely disabled] as not all readers may be familiar with the details of this scoring system. | Thanks for your important comment.  1a. We have now added the full name of ECOG in the abstract and in the table 1 legend as suggested.    1b. We have added the ECOG full statement as advised. The following text has been added in the method section of the revised manuscript  “(score of 0 = Fully active, score of 1= Restricted in physically strenuous activity, a score of 2= Ambulatory and capable of all self-care but unable to carry out any work activities, a score of 3= Capable of only limited self-care and score of 4= Completely disabled)”. |
| 2) The authors discuss that having a comorbidity also predicts poor outcome for lung cancer but need to give more clear details regarding the comorbidities that they assessed.  2a. In the Materials & Methods section, the authors state, “A patient was considered to have co morbidity if the patient has been suffering or receiving treatment for a major existing condition not related to the cancer or its complication (i.e., diabetes, hypertension, heart disease, or chronic obstructive pulmonary disease).” This sentence is confusing as currently worded. Were the above conditions what was considered a “comorbidity” or were these conditions considered to be related to cancer or its complication? If these are the diseases being considered then just say that these were considered the comorbidities.  2b. How did the authors determine the co-existence of the above comorbidities: was this based on chart review mentioning one of these diseases or was this based on the patient receiving medication to treat one of these diseases? Please clarify this important point.  2c. It appears that there were 4 comorbidities assessed for (if the above sentence is correct) and the authors appear to have assigned each subject a dichotomous “yes/no” for having a comorbidity. Did the authors do a separate analysis by the number of comorbidities for the n=551 who had at least 1 comorbidity. This would be an interesting analysis to see if having 2, 3 or 4 comorbidities conferred an even worse prognosis than just having 1. | Thanks for your valuable comment.  2a. The wording of the sentence we presented left the ground for ambiguity. We have rewritten the section to be clearer.  Following text has been added to the methods section of the revised manuscript  “A patient was considered to have co-morbidity if the patient had been suffering from or receiving treatment for a major existing condition such as, diabetes, hypertension, heart disease, or chronic obstructive pulmonary disease etc.”  2b. We agree with the author that, this is an important point. The following text has been added the method section of the revised manuscript.  “Comorbidity data of the patient were collected by trained data collector (physician) from the, chart review, current medication record, and cancer treatment eligibility check-up record conducted at the center.”    2c. Thanks for the insightful comment, we have conducted additional analysis according to the comment. We have added the following result in the result section of the revised manuscript.    “Around 29.5% (n=551) patients were reported to have at least one comorbidity, the comorbidities were Diabetes (9.0%), Hypertension (10.1%), COPD (11.9%), Asthma (1.0%), IHD (0.9%), Arthritis (1.7%). Out of the 551 patients with comorbidities, 461 had one comorbidity, 82 had two comorbidities and 7 patients had three or more comorbidities. Although presences of at least one co-morbidity is associated with lower survival. However, having multiple co-morbidity did not exert any additional synergistic effect on survival.” |
| 3)  Tobacco use status could benefit from some additional detail and analysis.  3a. In the Results section, the authors state that, ‘More than eighty percent of patients with lung cancer were smokers and more than half of the patients were smokeless tobacco users.”  In table 1, they separate out these 2 different tobaccos use categories. There is likely some overlap in the 2 groups which may be a synergistic risk factor (i.e., Both tobacco smoker and smokeless tobacco user). Could the authors provide the information on the number of subjects in the overlap group who used 2 types of tobacco. 3b. Did the authors do a separate analysis on those with both types of tobacco use to see if this was predictive of a poorer outcome? 3c. Could the authors provide information on the number of patients who were totally non-tobacco exposed (i.e., Not using smoking tobacco or smokeless tobacco) as this information is not able to be determined from Table 1. Was this protective in lung cancer? | Thanks for the insightful comment and we agree with the reviewer. We ran additional analysis and the following texts are added in the **result** section of the revised manuscript.  3a. “44.3% (n=827) patients consumed both the form of tobacco”.  3b. Consumption of any form of tobacco neither individual nor both are predictive of survival.  3c. “10.8% (208) persons were neither smoke tobacco nor smokeless tobacco user, their tobacco consumption status also did not seem to affect the survival” |
| 4) Grammatical and typographical errors: Throughout this manuscript there are numerous grammatical and typographical errors which need to be addressed prior to publication.  4a. Introduction, “…unveil useful insight regarding there link to…” The proper word is “their” not “there.”  4b. Materials & Methods section: There is a missing reference in the following sentence. “Body mass index (BMI) was categorized into …. And > 25 (overweight) (Ref).” Please provide the actual reference here.  4c. Discussion section, in the following sentence “accumulate” should not have an “s.” “…incidence of chronic disease like cancer are likely to be increased, patients are more likely to accumulates more risk exposure.”  4d. Discussion section: I the following sentence “female” should be pleural (i.e., “females”). “…for the indifference may lie in the smaller proportion of female in the study.”  4e. Discussion: in the following sentence the word “who” needs to be added in between “those had.” “…didn’t show any survival benefit over those had only primary level of education...”  4f. Discussion: The following sentence needs to be re-worded. “The impact on survival is probably, correlates more with the stage and severity of the disease, rather than by itself.”  4g. Discussion: There is a typographical error in the sentence below and should read as “218 and 2019.” Please replace the “1” with an “a.” “…attend the hospital during the year 2018 1nd 2019…”  5) Please consider standardizing the abstract.  5a. Statistics presented: For some variables the HR is given while for others no HR is given.  5b. Presentation of results associated with higher survival vs. lower survival. It would be more consistent and send a clearer message to give all of the factors associated with increased survival first and then present the variables associated with lower survival. | 4. Thank you so much for your thoughtful comments. We have corrected the following as suggested.  4a. “their”  4b. reference added.  4c. accumulate  4d. Females “s” added  4e. “who” added  4f. “Patients with low BMI are more likely to be of advanced stage, hence, the impact on survival among patient with low BMI is probably due to the stage and severity of the disease, rather than BMI itself.”  4g. and  5 (a.b). Thank you so much for your thoughtful comments. We have rewritten the abstract to standardize it as suggested, further the following texts have been included in the abstract of the revised manuscript  “Higher survival was associated with institutional education (HR 0.9; 95% CI 0.77, 0.99), and receipt of combined radiotherapy and chemotherapy (HR 0.56; 95% CI 0.46, 0.65; p <0.001). In contrast, lower survival was associated with older age between 60- 69 years (HR 1.3; 95% CI 1.3, 1.5;), age ≥ 70 years (HR 1.4; 95% CI 1.1, 1.7), having any comorbidity (HR 1.1; 95% CI 1.0, 1.3), with ECOG score ≥ 3 (HR 1.41; 95% CI 1.01, 1.96) and receipt of radiotherapy treatments only (HR 1.6; 95% CI 1.3, 1.9).” |
